# Diagnosis and treatment of breast cancer during pregnancy: a comprehensive review

**DOI:** 10.3389/fmed.2026.1811147

**Published:** 2026-04-22

**Authors:** Jiuxing Wei, Fengjiang Qu, Tinghan Jiang, Chengzhao Xu

**Affiliations:** 1 Department of Emergency Surgery, The First Hospital of Jilin University, Changchun, Jilin, China; 2 Hepatobiliary Pancreatic Surgery Department II, General Surgery Center, First Hospital of Jilin University, Changchun, China; 3 Department of Breast Surgery, General Surgery Center, The First Hospital of Jilin University, Changchun, Jilin, China

**Keywords:** breast cancer, diagnosis, evolution, pregnancy, treatment

## Abstract

Breast cancer during pregnancy (BCP) is clinically rare. Its occurrence during a unique physiological period introduces pregnancy-related complications that can seriously affect maternal health. Diagnostic and treatment criteria are lacking. Nowadays, breast cancer (BC) has surpassed lung cancer and become the most common malignant tumor in the world, posing a serious threat to women’s physical and mental health. BCP is a rare malignant tumor with unique characteristics for diagnosis and treatment. Diagnosing cancer at this particular time in a woman’s life is a challenge in clinical management because of the need to ensure psychological safety and pregnancy status. Lack of systematic diagnosis and treatment plans can affect patient outcomes. By reviewing the literature at home and abroad, we summarized the status of diagnosis and treatment of BCP as well as the controversial issues for clinical application, highlighting the evolutionary trajectory of management strategies in this field.

## 1 Introduction

Breast cancer (BC) is becoming the most common malignant tumor worldwide, a series of clinical studies have shown that the highest incidence of malignant tumors in pregnant women is breast cancer (1), seriously threatening women’s physical and mental health (2). BC during pregnancy (BCP) is a rare malignant tumor with unique features in diagnosis and treatment. The diagnosis of cancer during this particular period of a woman’s life is a challenge in clinical management because of the need to ensure psychological safety and pregnancy status (3). The lack of systematic diagnosis and treatment plan affects the prognosis of patients. A Canadian population-based cohort study found that pregnancy-associated cancers including BC had increased overall 5-years mortality (4). By reviewing domestic and foreign literature, we summarized the diagnosis and treatment status and controversial issues of BCP for reference in clinical application, with an emphasis on the evolution of diagnostic pathways and therapeutic paradigms over time.

## 2 Definition and epidemiology of BCP

Breast cancer during pregnancy refers to breast cancer diagnosed during pregnancy, which is a part of pregnancy-associated breast cancer (PABC). BCP has the second highest incidence of malignant tumors in women during pregnancy (5). PABC accounts for 0.2%−3.8% of breast malignancies (6, 7), including BCP and breast cancer occurring within 1 year after delivery (8). Evolving evidence supports the separation of BCP from postpartum BC (PPBC) due to their differences in incidence, pathological features and treatment (9). BCP has independent biological properties, so some scholars believe that the concept of PABC should not continue to be used (10). BCP accounts for about 4% of BC cases in women aged <45 years (11), and there is about 1 case in every 1000 pregnancies (12). In China, with the opening of the two-child policy and the emergence of delayed childbirth, the number and age of patients are gradually increasing (13).

## 3 Clinical features of BCP

Proper assessment of suspected BCP should include physical examination (with special attention to breast and regional lymph nodes), imaging examination, and mass histopathology. BCP patients can usually feel a painless lump in their breast or a painful lump which should be distinguished from mastitis during pregnancy. Due to the underlying anatomical and physiological changes in the breast during pregnancy: breast gland hyperplasia and volume enlargement, thus concealing the findings of some smaller lesions easily. In addition, the majority of patients are young women, and doctors are prone to subjectively consider benign tumors such as breast fibroadenomas, which causes BCP diagnosis is often delayed (14). During specialist physical examination, the breast mass is generally hard, with unclear boundaries, irregular shape, hard and fixation. There may also be inflammatory manifestations or nipple discharge (15, 16). Axillary lymph node enlargement can also be the first symptom. A small number of patients have irritating cough, hemoptysis and other respiratory disease symptoms as well as dizziness, fatigue, bone joint pain and central nervous system and bone metastasis (17, 18).

## 4 BCP diagnostic imaging examination

Ultrasound is still the standard first-line imaging examination of the breast, with high sensitivity, specificity and safety for the diagnosis of breast lesions (19). However, the diagnostic performance of breast ultrasound is not perfect, especially during pregnancy. A study by Robbins et al. reported that the sensitivity and specificity of breast ultrasound for detecting pregnancy-associated breast cancer were 86.4% and 96.5%, respectively (20). Despite its relatively high sensitivity, the specificity is far from 100%, leading to a potential for false positives. On ultrasound, BCP lesions can present as low or mixed echo masses with irregular shape and unclear boundaries. It has also been reported that some breast cancer foci during pregnancy present with the morphological characteristics of benign tumors with regular margins (21). The accuracy of ultrasound may be lower, but its advantages such as convenient operation and repeatability provide important clinical information, especially for the determination of breast lesions and axillary conditions, so it is still the preferred screening method at present (22, 23).

Mammography can be used to assess the degree of disease, identify atypical calcification, and assess the contralateral breast. Calcifications that cannot be imaged by ultrasound can be present, especially for dense mammary glands, with sensitivity typically between 78% and 90% (24). During pregnancy, due to concerns about the impact of radiation on fetal development, many patients resist this examination, but the radiation dose of bilateral mammography is <3 mGy, and the radiation dose to the uterus is <0.03 mGy, exposing the fetus to the minimum dose of radiation (0.001–0.01 mGy) (25, 26). Mammography is generally safe and feasible during pregnancy (27). While ultrasound is the initial imaging modality of choice, mammography may be particularly valuable as a complementary tool in specific populations, such as older pregnant patients (e.g., >35 years) or those with a prior history of mammographically detected abnormalities, where the evaluation of microcalcifications is crucial for diagnosis. For the lesions not found by breast ultrasound and X-ray examination, magnetic resonance imaging (MRI) is required to avoid the effects of radiation. Breast MRI without contrast agent is safe, but there is still insufficient evidence about the diagnostic value of MRI in BCP (28). Because gadolinium contrast agents can enter the fetal circulation and amniotic fluid through the blood–placenta barrier, the results in rats indicate that contrast-enhanced MRI is potentially teratogenic (29, 30). Therefore, routine breast MRI enhancement is not recommended for pregnant patients. However, the latest guidelines of the European Society of Urogenital Radiology for the use of contrast media in pregnant women suggest the use of gadolinium-based contrast media for breast MRI in pregnant women (31). Six radiologists from an Israeli Research Institute were selected for independent reading. Reduction in background-parenchymal-enhancement among PABC patients compared to the lactating controls suggests that several factors, including a possible vascular steal phenomenon, may affect the diagnostic rate in cancer patients (32). The author believes that under the current condition of insufficient evidence-based medical evidence, patients with BCP should be cautious when undergoing contrast-enhanced MRI.

Unnecessary radiography is not recommended during pregnancy for systemic assessment suspects metastasis. Abdominal ultrasound and chest X-ray examination under the protection of a lead barrier can be performed. If brain or bone metastases are suspected, MRI of the corresponding site can be performed (27). Nuclear medical examinations including bone scan and positron emission tomography are strictly contraindicated due to significant radionuclide radiation.

## 5 Pathological features of BCP

Pathological examination is still the gold standard for diagnosis. Like non-pregnant patients, biopsy and pathological examination should be completed in time for BI-RADS IV/V lesions with high clinical suspicion and imaging examination, so as to provide guidance for the next treatment plan. Under the conditions of excluding contraindications such as bleeding disorders and anesthetic allergies, Ultrasound-guided core needle biopsy is the preferred modality (33). During the puncture operation, attention should be paid to the depth and angle of needle insertion, and the substantial part of the mass should be selected for sampling. For multiple tumors, one lesion should correspond to one set of puncture instruments, and damage to important blood vessels, milk ducts and nerves should be avoided (29). When submitting the tumor tissue for pathological examination, pathologists should be reminded of the patient’s pregnancy, because the presence of proliferative cells may mimic atypia, leading to an increase in false-positive results. The most common histomorphological type is poorly differentiated invasive ductal carcinoma. BCP has been shown to be associated with lower hormone receptor expression rates and with more aggressive subtypes that are characteristic of younger cases (34), higher grade and proliferation rates, late T stage at diagnosis, lymph node involvement, higher prevalence of triple negative BC (TNBC) and hormone receptor negative subtypes (35, 36). Unlike non-pregnant women, these tumors usually involve axillary lymph nodes and blood vessels when found. It is noteworthy that approximately 30% of BCP cases are classified as TNBC, and 40% of cases have axillary lymph node infiltration. Small series of studies have suggested there were differences in gene expression between BCP and non-BCP, but no large study supports this theory (37). In a cohort study of 20 women diagnosed with BCP or in the first year after delivery, the pathogen mutation detection rate was 35% (7/20 cases). Among them, six patients had pathogenic BRCA1 mutations, and up to 30% of obviously high frequency BRCA1 mutations places BCP patients in a high risk environment (38, 39). Due to the high incidence of BRCA 1 or 2 mutations in young BC patients, genetic testing should be offered to women with BCP (40).

## 6 Evolution of local and systemic BCP treatment

The management of BCP has evolved significantly over the past decades, shifting from a historical tendency toward immediate pregnancy termination to a modern, multidisciplinary approach that aims to treat the cancer effectively while preserving the pregnancy. Management of BCP is complex due to the potential risks to the fetus during treatment. The optimal treatment strategy should be planned by a multidisciplinary team consisting of breast surgeons, oncologists, radiotherapists, gynecologists and neonatologists. The strategy should be designed to maximize treatment options for patients while minimizing potential adverse events for the fetus. Amant et al. suggest that therapeutic abortion is not routinely recommended, as breast cancer treatment can proceed alongside continued pregnancy. Human chorionic gonadotropin promotes cell differentiation, apoptosis, and growth inhibition in both mammary epithelial and breast cancer cells. A full-term pregnancy facilitates complete cell differentiation, while the abrupt hormonal decline following artificial abortion may increase cellular vulnerability. Consequently, pregnancy termination does not improve the patient’s prognosis (17). Termination of pregnancy does not improve patient prognosis and may have associated drawbacks. However, in cases of advanced tumors and poor prognosis during early pregnancy, abortion could be considered to prevent delays in diagnosis and treatment (17, 41). The guidelines suggest that BCP should be treated according to the same recommendations as for BC in young non-pregnant women (42, 43). Clinicopathological features, gestational age at diagnosis, due date, and patient selection are key factors for optimal treatment.

### 6.1 Surgical treatment

Surgery is one of the main treatment for BCP, and is considered relatively safe throughout pregnancy in the absence of other large underlying diseases and pregnancy-related complications with an acceptable range of trauma and should follow the same recommendations as for non-pregnant women when feasible (42). The fetal condition can be closely tracked from 24 to 26 weeks of gestation by intraoperative fetal heart rate monitoring (44). However, surgery in the first trimester is usually avoided if possible. All pregnant women undergoing surgery should take precautions to avoid uterine hypoperfusion, maternal hypotension, hypoxia, hypoglycemia, pain, fever and infection. Modified radical mastectomy is considered the standard treatment for BCP because it eliminates the need for postoperative radiotherapy and ultimately controls the axillary region, however, this kind of surgery has great physical and mental damage to young women. Therefore, some scholars have tried to perform breast conserving surgery on patients who meet the indications for breast conservation. Although there has not been a large specialized study, similar survival rates have been observed in women who receive breast-conserving or radical surgery. Small studies support the safety of staged reconstruction with tissue dilators placed immediately after mastectomy in BCP, and the benefits of immediate breast reconstruction include improved psychological and aesthetic outcomes (45). A series of women who received tissue dilators during pregnancy showed no increase in obstetric disease or surgical complications (46). In addition, anesthesia is an important factor to consider. Many physiological changes during pregnancy can affect anesthesia, and despite several preclinical and clinical observational studies, no significant risk to the fetus has been found from anesthetics, nor has the optimal anesthesia technique been identified. The potential negative effects of anesthetics on fetal development depend on the dose, duration of exposure associated with fetal development, and route of administration (24).

The availability of axillary sentinel lymph node biopsy (SLNB) in pregnant women has been controversial. The American Society of Clinical Oncology (ASCO) recommendation does not support this procedure, while the National Comprehensive Cancer Network (NCCN) guidelines do support this procedure based on several studies showing that it can be performed safely (47, 48). The main point of contention is the choice of tracer, Technetium 99m colloidal solution as the first choice is considered safe for SLNB by injection during pregnancy. Fetal radiation exposure after injection is within acceptable levels (49). An international cohort study involving 145 women with BCP reported high recognition rates and low axillary recurrence rates for SLNB (50). This approach is considered safe in patients with clinically node-negative BCP. Although technetium-99m has been shown to be safe, blue dyes are contraindicated because no studies have tested their safety. In particular, methylene blue has been reported to cause allergic reactions and fetal teratogenicity (51). Other tracers have not been well reported and are therefore not recommended for routine use.

### 6.2 Chemotherapy

Pregnancy has not been shown to be an absolute contraindication to the use of systemic chemotherapy to treat BC. The results of a large retrospective/prospective meta-analysis supporting the use of chemotherapy in pregnancy showed that intrauterine exposure to chemotherapy did not significantly increase the risk of fetal death or major congenital malformations compared with the general population (52). Therefore, a systematic treatment plan should be formulated according to the stages and biological characteristics of BC. The main concerns of chemotherapy in pregnancy are time, drug selection and measurement, it is also greatly related to the patients’ psychological acceptance of chemotherapy (53). Due to the potential impact of chemotherapy on fetal development, particularly during organogenesis (equivalent to 3–12 weeks of gestation), the risk of congenital malformations and fetal loss due to exposure to chemotherapy may be high. Retrospective data show that the incidence of major fetal malformations is 14%–20% when chemotherapy is administered early in pregnancy. The incidence of chemotherapy after the first trimester is 3%–5% (42). Therefore, neoadjuvant chemotherapy in the first trimester is contraindicated, but considered safe in the second and third trimesters (54). It is recommended to start chemotherapy at 14 weeks, which greatly reduces the body damage and fetal miscarriage rate (55). Delaying treatment until after delivery is not recommended, as it may lead to worse adverse outcomes (56). It is recommended to end chemotherapy before 36 weeks of gestation to avoid potential hematological complications during delivery (42, 57). Although BCPs reflect all subtypes of BC, they are most commonly invasive ductal carcinomas of high tumor grade and large tumor size, with more advanced stage at presentation and higher rates of lymph node involvement. Most BCPs are hormone negative tumors (triple negative or HER2 amplified tumors) with high Ki-67 proliferation rates (58). Regarding the selection of chemotherapy agents, anthracycline, cyclophosphamide and taxane are supported by international guidelines and are the standard for chemotherapy in BC (59–61). Physiological changes in pregnancy that affect systemic pharmacokinetics include increased maternal weight, plasma volume, liver and kidney perfusion, cytochrome P450 activity (third trimester), and changes in serum albumin concentration, and those under medication should be avoided. Therefore, the dose of chemotherapy should be calculated according to the actual body weight of the patient, and dose-intensive chemotherapy (same dose given at shorter intervals) can improve the therapeutic effect, especially in high-risk patients (42). A retrospective cohort study of 109 pregnant women with BC, 10 of whom received dose-intensive chemotherapy, demonstrated the safety of this approach (62). However, small studies limit the possibility of recommendations for the safety of dose-intensive chemotherapy in patients with BCP. Most prophylactic drugs commonly used in non-pregnant patients can also be safely administered during pregnancy. Histamine type 2 receptor antagonists can be used to prevent allergic reactions without increasing the risk of major malformations (63). A retrospective cohort study evaluating granulocyte colony-stimulating factor in pregnant women during chemotherapy found no difference in gestational age, congenital abnormalities, and neutropenia in exposed infants (64). In addition, the use of aprepitan, ondansetron, methylprednisolone and other supportive drugs during pregnancy is also considered safe (65). Anthracyclines are widely known for their cardiotoxic effects, depending on multiple mechanisms, including oxidative damage, changes in calcium metabolism, and activation of apoptotic pathways (66, 67). Although the use of chemotherapy in the second and third trimesters appears to be mostly safe and tolerated, a multidisciplinary approach, including rigorous fetal monitoring and maternal blood pressure control is needed. PPBC is more sensitive to chemotherapy than other patients. We need to pay more attention to this group and achieve individualized treatment, which will help us treat BC better (68).

### 6.3 Radiotherapy

Considering that radiation exposure can cause fetal malformations and other adverse events, it can cause miscarriage in the first trimester, and fetal neurological and reproductive system malformations after 3 weeks of pregnancy (69), and the fetal risk effect is also dose-related (70). In view of the above reports, radiotherapy for BCP is generally contraindicated. However, a report shows that ViTAT can be used in place of 3DCRT while maintaining similar fetal dose levels if 6XFFF beams are used, pregnant women and the fetus will be relatively safe (71). But if necessary, radiotherapy can be done after delivery.

### 6.4 Endocrine therapy

In animal models, tamoxifen has teratogenic effects, involving the urogenital tract more frequently, and increasing the risk of BC in offspring (72). A systematic review of pregnancy data for 248 BC patients exposed to tamoxifen during pregnancy showed a 17.6% incidence of severe malformation (73). Further studies suggest that tamoxifen can be excreted in breast milk and, therefore, must be avoided during lactation (74). International guidelines prohibit the use of tamoxifen during pregnancy and it should be discontinued immediately if the pregnancy is expected to continue.

### 6.5 Targeted therapy

In recent years, targeted therapies for BC have been used increasingly (75). A systematic review and meta-analysis to evaluate the safety of trastuzumab during pregnancy showed that the main adverse events were oligohydramnios and/or dehydration, which were related to gestational age and occurred in 73.3% of fetuses exposed in mid/late stages. This did not occur in fetuses with early exposure, and in subsequent follow-up, and fetuses exposed only in the early stages of pregnancy were also healthy after birth (76). Therefore, patients with early exposure to trastuzumab in small amounts may be able to continue pregnancy under close monitoring. Current guidelines contraindicate trastuzumab in patients with BCP due to its impact on fetal lung development and the occurrence of hydramnios during pregnancy (67), as well as unknown long-term consequences for the fetus. Pertuzumab, trastuzumab - entansine (T-DM1) and neratinib are in early use. Anne-Sophie Boudy et al. conducted a matched cohort study involving 102 patients with HER2-positive breast cancer and concluded that BCP patients exhibit poorer prognoses compared to non-pregnant patients (77).

## 7 Prognosis

Studies on prognosis of BCP are mostly small retrospective studies and survival analyses of PABC. Azim’s meta-analysis comparing 3,628 patients with PABC and 37,100 controls found that poor prognosis of PABC was mainly driven by patients with postpartum breast cancer rather than BC diagnosed during pregnancy (37). And recently, an American research institute Compared women who were diagnosed with PABC during gestation and those who were diagnosed within 12 months postpartum. While 10-years recurrence-free survival is similar between groups, 10-years overall survival is higher among women diagnosed postpartum, however, timing of diagnosis may not be the driving factor in determining survival outcomes (67, 78). A national study evaluating the difference in outcome between patients with BCP and PPBC showed no significant difference in 5-years overall survival rate and disease-free survival rate (19). Amant et al. reported comparable survival rates in patients diagnosed with BCP compared to patients without PABC (79). But Lundberg and Margioula-Siarkou think that preterm birth and related adverse outcomes were more common in women diagnosed with breast cancer during pregnancy (80, 81). Patients with PABC may have a poor prognosis, with a lower 5-years survival rate compared to age-matched general patients with BC, disease is more aggressive and has a poor prognosis, reducing delays in diagnosis and treatment may help to improve survival (82). Patients diagnosed with breast cancer during lactation have a worse prognosis compared to those with PBC, most likely due to disease progression rather than biological features (83). On systemic treatment, breastfeeding is not recommended because drug excretion may exist in breast milk (84). Whether pregnancy has an independent effect on the prognosis of BCP still needs to be investigated in a large-scale retrospective analysis (67, 85).

## 8 Conclusion

Breast cancer during pregnancy is a rare type of breast malignancy with unique pathological characteristics, and collaboration with obstetrics, neonatal and oncology departments is necessary due to the complexity of maternal treatment and potential impact on the fetus. Surgery is still one of the main treatment method, and the selection of chemotherapy period is important. Radiotherapy, endocrine therapy and targeted therapy are still contraindicated. After systematic treatment, good outcome and prognosis can be achieved.

## References

[1] Saad M Murphy M McGee S El-Chaâr D. Pregnancy and neonatal outcomes following malignancy in pregnancy at a tertiary care Canadian center: a retrospective chart review. *J Matern Fetal Neonatal Med.* (2023) 36:2198631. 10.1080/14767058.2023.2198631 37031968

[2] Sung H Ferlay J Siegel R Laversanne M Soerjomataram I Jemal A et al. Global cancer statistics 2020: globocan estimates of incidence and mortality worldwide for 36 cancers in 185 Countries. *CA Cancer J Clin.* (2021) 71:209–49. 10.3322/caac.21660 33538338

[3] Kyriazoglou A Korakiti A Papatheodoridi A Apostolidou K Nonni A Zografos E et al. Chasing hippos: implications of YAP1 and TAZ Expression in pregnancy-associated breast cancer tumorigenesis. *In Vivo.* (2022) 36:2869–74. 10.21873/invivo.13027 36309401 PMC9677786

[4] Cairncross Z Shack L Nelson G Friedenreich C Ray J Fell D et al. Long-term mortality in individuals diagnosed with cancer during pregnancy or postpartum. *JAMA Oncol.* (2023) 9:791–9. 10.1001/jamaoncol.2023.0339 37022714 PMC10080404

[5] Maxwell C Al-Sehli H Parrish J D’Souza R. Breast cancer in pregnancy: a retrospective cohort study. *Gynecol Obstet Invest.* (2019) 84:79–85. 10.1159/000493128 30219806

[6] Lee Y Roberts C Dobbins T Stavrou E Black K Morris J et al. Incidence and outcomes of pregnancy-associated cancer in Australia, 1994-2008: a population-based linkage study. *BJOG.* (2012) 119:1572–82. 10.1111/j.1471-0528.2012.03475.x 22947229 PMC3533794

[7] Balasch J Gratacós E. Delayed childbearing: effects on fertility and the outcome of pregnancy. *Curr Opin Obstet Gynecol.* (2012) 24:187–93. 10.1097/GCO.0b013e3283517908 22450043

[8] Prior L O’Dwyer R Farooq A Greally M Ward C O’Leary C et al. Pregnancy-associated breast cancer: evaluating maternal and foetal outcomes. A national study. *Breast Cancer Res Treat.* (2021) 189:269–83. 10.1007/s10549-021-06263-y 34125341

[9] Barreau L Gau S Loussert L Vaysse C Weyl A Groussolles M. [Cancer during pregnancy: proposal of a clinical care pathway based on a regional cohort]. *Gynecol Obstet Fertil Senol.* (2022) 50:657–65. 10.1016/j.gofs.2022.07.003 35843588

[10] Amant F Lefrère H Borges V Cardonick E Lambertini M Loibl S et al. The definition of pregnancy-associated breast cancer is outdated and should no longer be used. *Lancet Oncol.* (2021) 22:753–4. 10.1016/S1470-2045(21)00183-2 34087122 PMC8868503

[11] Callihan E Gao D Jindal S Lyons T Manthey E Edgerton S et al. Postpartum diagnosis demonstrates a high risk for metastasis and merits an expanded definition of pregnancy-associated breast cancer. *Breast Cancer Res Treat.* (2013) 138:549–59. 10.1007/s10549-013-2437-x 23430224 PMC3608871

[12] Dalmartello M Negri E La Vecchia C Scarfone G Buonomo B Peccatori F et al. Frequency of pregnancy-associated cancer: a systematic review of population-based studies. *Cancers.* (2020) 12:1356. 10.3390/cancers12061356 32466494 PMC7352408

[13] Lee G Mayer E Partridge A. Prognosis of pregnancy-associated breast cancer. *Breast Cancer Res Treat.* (2017) 163:417–21. 10.1007/s10549-017-4224-6 28365832

[14] Vashi R Hooley R Butler R Geisel J Philpotts L. Breast imaging of the pregnant and lactating patient: imaging modalities and pregnancy-associated breast cancer. *AJR Am J Roentgenol.* (2013) 200:321–8. 10.2214/ajr.12.9814 23345353

[15] Macdonald H. Pregnancy associated breast cancer. *Breast J.* (2020) 26:81–5. 10.1111/tbj.13714 31943583

[16] Girardelli S Rabaiotti E Mauro F Gentilini O Zucchinelli P Cioffi R et al. Weekly paclitaxel administered during a twin pregnancy for recurrent breast cancer: case report and review of the literature. *J Adolesc Young Adult Oncol.* (2022) 11:632–6. 10.1089/jayao.2021.0181 35180353

[17] Amant F Loibl S Neven P Van Calsteren K. Breast cancer in pregnancy. *Lancet.* (2012) 379:570–9. 10.1016/S0140-6736(11)61092-1 22325662

[18] Rossi L Mazzara C Pagani O. Diagnosis and treatment of breast cancer in young women. *Curr Treat Options Oncol.* (2019) 20:86. 10.1007/s11864-019-0685-7 31776799

[19] Matos E Ovcaricek T. Breast cancer during pregnancy: retrospective institutional case series. *Radiol Oncol.* (2021) 55:362–8. 10.2478/raon-2021-0022 33939895 PMC8366736

[20] Robbins J Jeffries D Roubidoux M Helvie M. Accuracy of diagnostic mammography and breast ultrasound during pregnancy and lactation. *AJR Am J Roentgenol.* (2011) 196:716–22. 10.2214/AJR.09.3662 21343518

[21] Jafari M Abbasvandi F Nazeri E Olfatbakhsh A Kaviani A Esmaeili R. Ultrasound features of pregnancy-associated breast cancer: a retrospective observational analysis. *Cancer Med.* (2023) 12:1189–94. 10.1002/cam4.4974 35748020 PMC9883397

[22] Reyes E Xercavins N Saura C Espinosa-Bravo M Gil-Moreno A Cordoba O. Breast cancer during pregnancy: matched study of diagnostic approach, tumor characteristics, and prognostic factors. *Tumori.* (2020) 106:378–87. 10.1177/0300891620925158 32623975

[23] Qian Y Chang C Zhang H. Ultrasound imaging characteristics of breast lesions diagnosed during pregnancy and lactation. *Breastfeed Med.* (2019) 14:712–7. 10.1089/bfm.2019.0155 31539269

[24] Paris I Di Giorgio D Carbognin L Corrado G Garganese G Franceschini G et al. Pregnancy-associated breast cancer: a multidisciplinary approach. *Clin Breast Cancer.* (2021) 21:e120–7. 10.1016/j.clbc.2020.07.007 32778512

[25] Wang P Chong S Kielar A Kelly A Knoepp U Mazza M et al. Imaging of pregnant and lactating patients: part 2, evidence-based review and recommendations. *AJR Am J Roentgenol.* (2012) 198:785–92. 10.2214/AJR.11.8223 22451542

[26] Lund P Saltvig I Oldenburg M Matzen S. [Diagnostics, treatment and prognosis in breast cancer during pregnancy]. *Ugeskr Laeger.* (2018) 180:V09170665. 29984695

[27] Obstetrics Gynecology. Committee opinion No. 723: guidelines for diagnostic imaging during pregnancy and lactation: correction. *Obstet Gynecol.* (2018) 132:786. 10.1097/AOG.0000000000002858 30134410

[28] Lother D Robert M Elwood E Smith S Tunariu N Johnston S et al. Imaging in metastatic breast cancer, CT, PET/CT, MRI, WB-DWI, CCA: review and new perspectives. *Cancer Imaging.* (2023) 23:53. 10.1186/s40644-023-00557-8 37254225 PMC10230813

[29] Shah N Scott D Kandagatla P Moravek M Cobain E Burness M et al. Young women with breast cancer: fertility preservation options and management of pregnancy-associated breast cancer. *Ann Surg Oncol.* (2019) 26:1214–24. 10.1245/s10434-019-07156-7 30680478 PMC6458084

[30] Osei E Darko J. Foetal radiation dose and risk from diagnostic radiology procedures: a multinational study. *ISRN Radiol.* (2013) 2013:318425. 10.5402/2013/318425 24959554 PMC4045527

[31] Thomsen H. Contrast media safety-an update. *Eur J Radiol.* (2011) 80:77–82. 10.1016/j.ejrad.2010.12.104 21856102

[32] Nissan N Massasa E Bauer E Halshtok-Neiman O Shalmon A Gotlieb M et al. MRI can accurately diagnose breast cancer during lactation. *Eur Radiol.* (2023) 33:2935–44. 10.1007/s00330-022-09234-z 36348090

[33] Jahanbin B Soleimani V. Histology of pregnancy-associated breast cancer. *Adv Exp Med Biol.* (2020) 1252:81–6. 10.1007/978-3-030-41596-9_10 32816265

[34] Perachino M Poggio F Arecco L Blondeaux E Spinaci S Marrocco C et al. Update on pregnancy following breast cancer diagnosis and treatment. *Cancer J.* (2022) 28:176–82. 10.1097/PPO.0000000000000599 35594464

[35] Poggio F Tagliamento M Pirrone C Soldato D Conte B Molinelli C et al. Update on the management of breast cancer during pregnancy. *Cancers.* (2020) 12:3616. 10.3390/cancers12123616 33287242 PMC7761659

[36] Peccatori F Lambertini M Scarfone G Del Pup L Codacci-Pisanelli G. Biology, staging, and treatment of breast cancer during pregnancy: reassessing the evidences. *Cancer Biol Med.* (2018) 15:6–13. 10.20892/j.issn.2095-3941.2017.0146 29545964 PMC5842335

[37] Azim H Brohée S Peccatori F Desmedt C Loi S Lambrechts D et al. Biology of breast cancer during pregnancy using genomic profiling. *Endocr Relat Cancer.* (2014) 21:545–54. 10.1530/ERC-14-0111 24825746

[38] Murphy C Mallam D Stein S Patil S Howard J Sklarin N et al. Current or recent pregnancy is associated with adverse pathologic features but not impaired survival in early breast cancer. *Cancer.* (2012) 118:3254–9. 10.1002/cncr.26654 22086863

[39] Zografos E Korakiti A Andrikopoulou A Rellias I Dimitrakakis C Marinopoulos S et al. Germline mutations in a clinic-based series of pregnancy associated breast cancer patients. *BMC Cancer.* (2021) 21:572. 10.1186/s12885-021-08310-9 34011307 PMC8132440

[40] Bobbili P Olufade T DerSarkissian M Shenolikar R Yu H Duh M et al. Adherence to national comprehensive cancer network guidelines for BRCA testing among high risk breast cancer patients: a retrospective chart review study. *Hered Cancer Clin Pract.* (2020) 18:13. 10.1186/s13053-020-00144-z 32518611 PMC7275608

[41] Schüler-Toprak S Treeck O Ortmann O. Human chorionic gonadotropin and breast cancer. *Int J Mol Sci.* (2017) 18:1587. 10.3390/ijms18071587 28754015 PMC5536074

[42] Peccatori F Azim H Orecchia R Hoekstra H Pavlidis N Kesic V et al. Cancer, pregnancy and fertility: ESMO clinical practice guidelines for diagnosis, treatment and follow-up. *Ann Oncol.* (2013) 24:vi160–70. 10.1093/annonc/mdt199 23813932

[43] Loibl S Schmidt A Gentilini O Kaufman B Kuhl C Denkert C et al. Breast cancer diagnosed during pregnancy: adapting recent advances in breast cancer care for pregnant patients. *JAMA Oncol.* (2015) 1:1145–53. 10.1001/jamaoncol.2015.2413 26247818

[44] Galati F Magri V Arias-Cadena P Moffa G Rizzo V Pasculli M et al. Pregnancy-associated breast cancer: a diagnostic and therapeutic challenge. *Diagnostics.* (2023) 13:604. 10.3390/diagnostics13040604 36832092 PMC9955856

[45] Toesca A Gentilini O Peccatori F Azim H Amant F. Locoregional treatment of breast cancer during pregnancy. *Gynecol Surg.* (2014) 11:279–84. 10.1007/s10397-014-0860-6 25419205 PMC4237906

[46] Lohsiriwat V Peccatori F Martella S Azim H Sarno M Galimberti V et al. Immediate breast reconstruction with expander in pregnant breast cancer patients. *Breast.* (2013) 22:657–60. 10.1016/j.breast.2013.06.005 23871328

[47] Lyman G Somerfield M Giuliano A. Sentinel lymph node biopsy for patients with early-stage breast cancer: 2016 american society of clinical oncology clinical practice guideline update summary. *J Oncol Pract.* (2017) 13:196–8. 10.1200/JOP.2016.019992 28118104

[48] Gradishar W Anderson B Abraham J Aft R Agnese D Allison K et al. Breast cancer, version 3.2020, NCCN clinical practice guidelines in oncology. *J Natl Compr Canc Netw.* (2020) 18:452–78. 10.6004/jnccn.2020.0016 32259783

[49] Gropper A Calvillo K Dominici L Troyan S Rhei E Economy K et al. Sentinel lymph node biopsy in pregnant women with breast cancer. *Ann Surg Oncol.* (2014) 21:2506–11. 10.1245/s10434-014-3718-2 24756813

[50] Han S Amant F Cardonick E Loibl S Peccatori F Gheysens O et al. Axillary staging for breast cancer during pregnancy: feasibility and safety of sentinel lymph node biopsy. *Breast Cancer Res Treat.* (2018) 168:551–7. 10.1007/s10549-017-4611-z 29235045

[51] Pruthi S Haakenson C Brost B Bryant K Reid J Singh R et al. Pharmacokinetics of methylene blue dye for lymphatic mapping in breast cancer-implications for use in pregnancy. *Am J Surg.* (2011) 201:70–5. 10.1016/j.amjsurg.2009.03.013 21167367

[52] de Haan J Verheecke M Van Calsteren K Van Calster B Shmakov R Mhallem Gziri M et al. Oncological management and obstetric and neonatal outcomes for women diagnosed with cancer during pregnancy: a 20-year international cohort study of 1170 patients. *Lancet Oncol.* (2018) 19:337–46. 10.1016/S1470-2045(18)30059-7 29395867

[53] Oprean C Ciocoiu A Segarceanu N Moldoveanu D Stan A Hoinoiu T et al. Pregnancy in a young patient with metastatic HER2-positive breast cancer-between fear of recurrence and desire to procreate. *Curr Oncol.* (2023) 30:4833–43. 10.3390/curroncol30050364 37232822 PMC10217573

[54] Barzen G Stangl K Blohmer J Henrich W Dörner T Lembcke A et al. Pregnancy and breast cancer in a patient with complicated Kawasaki disease, as if one problem was not enough: a case report. *Eur Heart J Case Rep.* (2022) 6:ytac435. 10.1093/ehjcr/ytac435 36451807 PMC9704422

[55] Amant F Berveiller P Boere I Cardonick E Fruscio R Fumagalli M et al. Gynecologic cancers in pregnancy: guidelines based on a third international consensus meeting. *Ann Oncol.* (2019) 30:1601–12. 10.1093/annonc/mdz228 31435648

[56] National Toxicology Program. NTP monograph: developmental effectsand pregnancy outcomes associated with cancer chemotherapy use duringpregnancy. *NTP Monogr.* (2013) 2:i–214. 24736875

[57] Bajpai J Pathak R Shylasree T Rugo H. Management of breast cancer diagnosed during pregnancy: global perspectives. *Expert Rev Anticancer Ther.* (2022) 22:1301–8. 10.1080/14737140.2022.2150167 36480337

[58] Proussaloglou E Blanco L Siziopikou K. Updates in the pathology of pregnancy associated breast cancer (PABC). *Pathol Res Pract.* (2023) 244:154413. 10.1016/j.prp.2023.154413 36921545

[59] Cardoso F Kyriakides S Ohno S Penault-Llorca F Poortmans P Rubio I et al. Early breast cancer: ESMO clinical practice guidelines for diagnosis, treatment and follow-up†. *Ann Oncol.* (2019) 30:1194–220. 10.1093/annonc/mdz173 31161190

[60] Denduluri N Somerfield M Giordano S. Selection of optimal adjuvant chemotherapy and targeted therapy for early breast cancer: ASCO clinical practice guideline focused update summary. *J Oncol Pract.* (2018) 14:508–10. 10.1200/JOP.18.00207 29924666

[61] Zagouri F Sergentanis T Chrysikos D Dimitrakakis C Tsigginou A Zografos C et al. Taxanes for breast cancer during pregnancy: a systematic review. *Clin Breast Cancer.* (2013) 13:16–23. 10.1016/j.clbc.2012.09.014 23122538

[62] Cardonick E Gilmandyar D Somer R. Maternal and neonatal outcomes of dose-dense chemotherapy for breast cancer in pregnancy. *Obstet Gynecol.* (2012) 120:1267–72. 10.1097/aog.0b013e31826c32d9 23168749

[63] Garbis H Elefant E Diav-Citrin O Mastroiacovo P Schaefer C Vial T et al. Pregnancy outcome after exposure to ranitidine and other H2-blockers. A collaborative study of the European network of teratology information services. *Reprod Toxicol.* (2005) 19:453–8. 10.1016/j.reprotox.2004.09.002 15749258

[64] Dale D Cottle T Fier C Bolyard A Bonilla M Boxer L et al. Severe chronic neutropenia: treatment and follow-up of patients in the Severe chronic neutropenia international registry. *Am J Hematol.* (2003) 72:82–93. 10.1002/ajh.10255 12555210

[65] Amant F Deckers S Van Calsteren K Loibl S Halaska M Brepoels L et al. Breast cancer in pregnancy: recommendations of an international consensus meeting. *Eur J Cancer.* (2010) 46:3158–68. 10.1016/j.ejca.2010.09.010 20932740

[66] Sawyer D Peng X Chen B Pentassuglia L Lim C. Mechanisms of anthracycline cardiac injury: can we identify strategies for cardioprotection? *Prog Cardiovasc Dis.* (2010) 53:105–13. 10.1016/j.pcad.2010.06.007 20728697 PMC2933091

[67] Sullivan E Safi N Li Z Remond M Chen T Javid N et al. Perinatal outcomes of women with gestational breast cancer in Australia and New Zealand: a prospective population-based study. *Birth.* (2022) 49:763–73. 10.1111/birt.12642 35470904 PMC9790712

[68] Dou H Jia S Ba Y Luo D Yu P Li F et al. Clinical characteristics and pathologic complete response (pCR) rate after neoadjuvant chemotherapy in postpartum women with breast cancer. *J Cancer Res Clin Oncol.* (2023) 149:14185–204. 10.1007/s00432-023-05194-z 37555951 PMC10590317

[69] Andersson T Johansson A Hsieh C Cnattingius S Lambe M. Increasing incidence of pregnancy-associated breast cancer in Sweden. *Obstet Gynecol.* (2009) 114:568–72. 10.1097/AOG.0b013e3181b19154 19701036

[70] Cardonick E Iacobucci A. Use of chemotherapy during human pregnancy. *Lancet Oncol.* (2004) 5:283–91. 10.1016/S1470-2045(04)01466-4 15120665

[71] Dusi F Guida F Garcia E Rossato M Germani A Sapignoli S et al. Fetal dose estimation for virtual tangential-fields arc therapy whole breast irradiation by optically stimulated luminescence dosimeters. *Phys Med.* (2022) 101:44–9. 10.1016/j.ejmp.2022.07.007 35944444

[72] Barthelmes L Gateley C. Tamoxifen and pregnancy. *Breast.* (2004) 13:446–51. 10.1016/j.breast.2004.08.007 15563850

[73] Buonomo B Brunello A Noli S Miglietta L Del Mastro L Lambertini M et al. Tamoxifen exposure during pregnancy: a systematic review and three more cases. *Breast Care.* (2020) 15:148–56. 10.1159/000501473 32398983 PMC7204783

[74] Peccatori F Codacci-Pisanelli G Mellgren G Buonomo B Baldassarre E Lien E et al. First-in-human pharmacokinetics of tamoxifen and its metabolites in the milk of a lactating mother: a case study. *ESMO Open.* (2020) 5:e000859. 10.1136/esmoopen-2020-000859 33115771 PMC7594360

[75] Lambertini M Peccatori F Azim H. Targeted agents for cancer treatment during pregnancy. *Cancer Treat Rev.* (2015) 41:301–9. 10.1016/j.ctrv.2015.03.001 25795021

[76] Zagouri F Sergentanis T Chrysikos D Papadimitriou C Dimopoulos M Bartsch R. Trastuzumab administration during pregnancy: a systematic review and meta-analysis. *Breast Cancer Res Treat.* (2013) 137:349–57. 10.1007/s10549-012-2368-y 23242615

[77] Boudy A Ferrier C Selleret L Zilberman S Arfi A Sussfeld J et al. Prognosis of HER2-positive pregnancy-associated breast cancer: analysis from the French CALG (Cancer Associé à La Grossesse) network. *Breast.* (2020) 54:311–8. 10.1016/j.breast.2020.11.013 33271423 PMC7711283

[78] Crown A McCartan D Curry M Patil S Kamer S Goldfarb S et al. Pregnancy-associated breast cancer: does timing of presentation affect outcome? *Breast Cancer Res Treat.* (2023) 198:283–94. 10.1007/s10549-022-06833-8 36662395

[79] Amant F von Minckwitz G Han S Bontenbal M Ring A Giermek J et al. Prognosis of women with primary breast cancer diagnosed during pregnancy: results from an international collaborative study. *J Clin Oncol.* (2013) 31:2532–9. 10.1200/JCO.2012.45.6335 23610117

[80] Lundberg F Gkekos L Rodriguez-Wallberg K Fredriksson I Johansson A. Risk of obstetric and perinatal complications in women presenting with breast cancer during pregnancy and the first year postpartum in Sweden 1973-2017: a population-based matched study. *Acta Obstet Gynecol Scand.* (2024) 103:684–94. 10.1111/aogs.14555 36959086 PMC10993363

[81] Margioula-Siarkou G Margioula-Siarkou C Petousis S Vavoulidis E Margaritis K Almperis A et al. Breast carcinogenesis during pregnancy: molecular mechanisms, maternal and fetal adverse outcomes. *Biology.* (2023) 12:408. 10.3390/biology12030408 36979100 PMC10045536

[82] Tan Q Alcantara V Sultana R Loh K Go A Wong F. Pregnancy-associated breast cancer: a multicenter study comparing clinicopathological factors, diagnosis and treatment outcomes with non-pregnant patients. *Breast Cancer Res Treat.* (2023) 198:53–66. 10.1007/s10549-022-06855-2 36617357

[83] Kataoka A Ueno T Yamauchi H Uehiro N Takahata C Takahashi Y et al. Characteristics, treatment trends, and long-term outcomes of Japanese patients with pregnancy-associated breast cancer (PABC). *Breast Cancer.* (2022) 29:825–34. 10.1007/s12282-022-01362-0 35604614

[84] Peccatori F Migliavacca Zucchetti B Buonomo B Bellettini G Codacci-Pisanelli G Notarangelo M. Lactation during and after breast cancer. *Adv Exp Med Biol.* (2020) 1252:159–63. 10.1007/978-3-030-41596-9_22 32816277

[85] Shao C Yu Z Xiao J Liu L Hong F Zhang Y et al. Prognosis of pregnancy-associated breast cancer: a meta-analysis. *BMC Cancer.* (2020) 20:746. 10.1186/s12885-020-07248-8 32778072 PMC7418189

